# Mechanistic Insights into PFAS Rejection in Nanofiltration and Reverse Osmosis from Data-Driven Analysis

**DOI:** 10.1021/acs.est.6c02287

**Published:** 2026-04-30

**Authors:** Yukai Tomsovic, Siwei Gu, Kyle Doudrick, Anthony P. Straub

**Affiliations:** † Department of Mechanical and Process Engineering, 1877 ETH Zürich, Zürich 8092, Switzerland; ‡ Materials Science and Engineering Program, University of Colorado Boulder, Boulder, Colorado 80309-0428, United States; § Department of Civil and Environmental Engineering and Earth Sciences, 6111 University of Notre Dame, Notre Dame, Indiana 46556, United States; ∥ Department of Civil, Environmental and Architectural Engineering, University of Colorado Boulder, Boulder, Colorado 80309-0428, United States

**Keywords:** per- and polyfluoroalkyl substances, reverse osmosis, nanofiltration, meta-analysis, machine learning

## Abstract

The structural diversity and complex transport behavior of per- and polyfluoroalkyl substances (PFAS) complicate a universal characterization of their removal in membrane systems. This study compiles 2353 data points from the literature on PFAS rejection by nanofiltration and reverse osmosis membranes, spanning a broad range of PFAS, membranes, feedwater compositions, and operating conditions. Using machine learning, this data set is modeled to evaluate how solute, membrane, and solution properties jointly influence PFAS removal. Of the 13 experimental system descriptors analyzed, membrane water permeance and PFAS molecular volume demonstrated the strongest main effect on rejection, emphasizing the significance of steric exclusion. The effects of background ions and dissolved organic matter were highly condition-dependent, exhibiting nonmonotonic behavior governed by competing mechanisms. Low concentrations of organic matter and ions enhanced PFAS rejection, consistent with complexation that increases apparent solute size, while higher concentrations reduced PFAS rejection, indicating that charge shielding and concentration polarization increasingly drive transport behavior. Overall, this analysis provides a unified, data-driven framework for interpreting previously inconsistent findings across studies, identifies critical gaps in existing experimental data, and highlights opportunities to guide targeted membrane design and treatment strategies.

## Graphical abstract

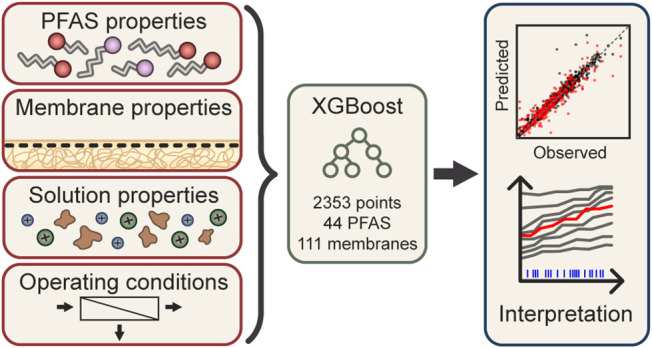

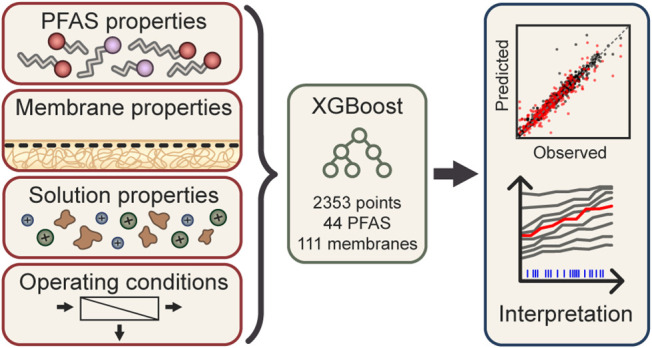

## Introduction

Per- and polyfluoroalkyl substances (PFAS) are a class of synthetic fluorinated compounds that have become ubiquitous in the environment due to their resistance to degradation and widespread use in industrial processes and consumer products.^1,2^ The accumulation of PFAS in the bloodstream has been linked to a range of adverse health effects, including immune and respiratory toxicity, endocrine disruption, and reproductive harm, underscoring the urgent need to remediate PFAS-impacted sites. Contaminated water sources represent a major route of human PFAS exposure, both through direct consumption and indirectly via bioaccumulation in aquatic organisms and agricultural products.^3,4^ However, current destruction technologies to remove PFAS from water are often limited by high energy and cost requirements.^5,6^ Membrane technologies, such as reverse osmosis (RO) and nanofiltration (NF), offer a promising approach to remove PFAS from contaminated water and effectively concentrate PFAS, making subsequent destruction more energy efficient and manageable.^7–9^ Thus, membranes may serve as a critical treatment step in the overall PFAS management strategy.

In response to the urgency of PFAS remediation, research on membrane-based PFAS removal has grown rapidly. Studies have explored diverse membrane materials, feed compositions, and PFAS chemistries to guide the design of improved membrane systems for PFAS removal.^10^ These investigations have identified the significance of steric, electrostatic, and hydrophobic interactions in governing PFAS rejection. For example, membrane rejection declines with decreasing PFAS chain length due to reduced steric hindrance while more negative membrane charge enhances repulsion of anionic PFAS.^11,12^ Coexisting salts and natural organic matter can further alter how these rejection mechanisms combine to determine membrane performance.^13,14^ Given the structural diversity of PFAS and the wide range of experimental conditions under which their removal is examined, it remains challenging to compare results across studies and apply findings to different treatment scenarios. Thus, there is a need for data-driven methods to synthesize and interpret PFAS rejection behavior.

Machine learning has emerged as a powerful tool for analyzing complex relationships in membrane processes and predicting solute rejection. In studies on organic micropollutant removal, machine learning models outperform traditional regression approaches, achieving high predictive accuracy across diverse solute types and membrane systems.^15–17^ These successes demonstrate the potential of data-driven methods to advance PFAS research, where the structural diversity and complex transport behavior of PFAS require flexible models capable of capturing nonlinear and interacting effects. Singh et al. and Hosseinzadeh et al. applied machine learning models with literature-derived data sets to accurately predict perfluorooctanesulfonic acid (PFOS) rejection, while Jeong et al. expanded this approach to multiple PFAS to evaluate the influence of molecular properties.^18–20^ However, these models were limited to either a single PFAS or a narrow range of feedwater conditions, leaving a critical gap in the quantitative understanding of how solute, membrane, and feedwater characteristics jointly govern PFAS rejection.

To enable a more comprehensive analysis of PFAS transport, we assembled the largest data set to date on PFAS rejection in NF and RO membranes, spanning a broad range of PFAS chemistries, membrane structures, and feedwater conditions (Figure 1). Leveraging this data set, we trained an extreme gradient boosting (XGBoost) model to evaluate the key factors determining PFAS rejection. We employed model interpretability techniques, including feature importance metrics and visualization of model predictions, to uncover nonlinear trends and quantify interactive effects among solute, membrane, and solution variables. By synthesizing results across diverse studies, our analysis also highlights underexplored areas in the literature, yielding actionable insights to guide future PFAS treatment research. Ultimately, this study establishes a data-driven framework to elucidate PFAS rejection mechanisms and inform the design of next-generation membrane systems for PFAS remediation.

**Figure 1:**
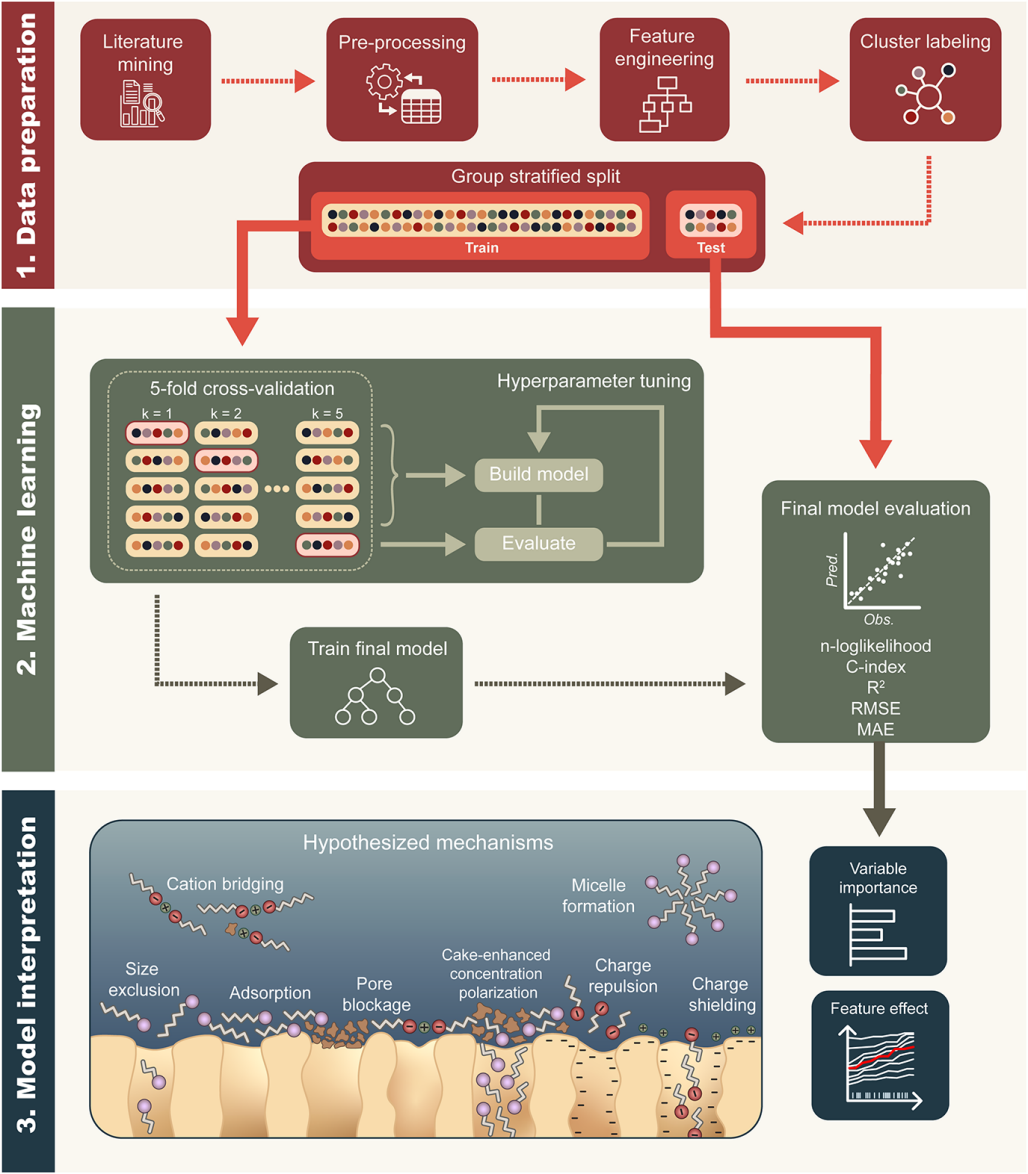
Diagram of machine learning workflow, including methods for interpretation.

## Methods

### Literature Mining and Data Collection

A total of 60 studies testing PFAS rejection with commercial and lab-synthesized RO or NF membranes were analyzed. From the selected studies, 2353 data points were manually obtained from tables and the text or extracted from figures using a web-based tool (PlotDigitizer). Rejection values reported at the limit of detection were flagged as right censored. Corresponding experimental conditions, including membrane properties, feedwater chemistry, operating conditions, and PFAS physicochemical properties were also recorded. For studies of commercial membranes without in-study characterization of membrane properties, relevant data were obtained from supplementary sources. PFAS physicochemical properties were calculated using Chemicalize (ChemAxon Marvin). A statistical summary of all variables collected is provided in Table S1, and the complete source data set is available online (see Supporting Information).

### Data Preprocessing and Splitting

A logit transformation (eq 1) was applied to correct the left-skew distribution of rejection values and to better model logarithmic differences in PFAS rejection, *R*.

1 $$\log it\left(R\right)=\ln\left(\frac{R}{1-R}\right)$$

As part of preprocessing, outliers with PFAS rejection below 0.50 were excluded to focus the analysis on higher rejection ranges more practically relevant for water treatment. K-means clustering was used to partition the data set into groups with similar experimental conditions by minimizing within-cluster variance.^21^ The optimal number of clusters was determined using the elbow method on membrane, PFAS, and feed composition features. The resulting cluster labels were then used to perform a stratified cluster split, dividing the data set into 85% training and validation and 15% test sets to ensure a well-balanced and representative distribution of experimental conditions across both subsets for robust model evaluation. Data points were stratified across clusters rather than grouped strictly by reference to preserve variability across experimental conditions. Although splitting by reference could avoid potential leakage, this approach would have resulted in reduced diversity and balance across splits. Importantly, the preprocessing steps used (e.g., logit transformation) do not rely on training-set statistics, minimizing data leakage risk. Figure S1 shows the distribution of rejection values in the training and test data sets before and after transformation.

### Feature Engineering and Selection

To reduce dimensionality, elemental feed composition was grouped by anion and cation ion valency. For studies that reported individual model foulant concentrations, total DOC was estimated using conversion factors: 50% of humic acid, 36% of sodium alginate, and 53% of bovine serum albumin mass concentrations. Spearman correlation coefficients were calculated between all features and the response variable. In cases of multicollinearity, only the feature with the highest correlation to the response was retained for modeling, while the others were excluded. Membrane properties with insufficient data coverage (e.g., root-mean-square roughness with >60% missing values) or high variability in reported measurements across studies, such as water contact angle, were also excluded. Based on this process, the final model included 13 features spanning four categories: membrane properties (pure water permeance, oxygen:nitrogen ratio, pH), PFAS physicochemical characteristics (van der Waals volume, dipole moment, p*K*_a_), operating conditions (temperature, permeate flux, PFAS feed concentration), and water chemistry (monovalent cation, divalent cation, trivalent cation, and dissolved organic carbon concentrations). All features extracted, including those excluded from analysis, are provided in the Supporting Information to support use in future research.

### Model Development and Evaluation

An XGBoost accelerated failure time (AFT) model was developed to accommodate right-censored PFAS rejection values that reached analytical detection limits. XGBoost, an extreme gradient boosting algorithm, was selected for its ability to capture nonlinear relationships, handle missing data natively, and model complex interactions among features with high computational efficiency. The model was trained using negative log-likelihood as the objective function. Hyperparameters were optimized using Bayesian optimization with a tree-structured Parzen Estimator (TPE) implemented via Optuna.^22^ 5-fold cross-validation with stratified splits based on data clusters was used during hyperparameter tuning to ensure balanced representation of experimental conditions in each fold. The following hyperparameters were tuned: learning rate, AFT loss distribution type and scale, maximum tree depth, subsample, column sample by tree, minimum child weight, lambda, alpha, and number of estimators. Model performance was also evaluated using concordance index (C-index), a ranking-based metric that accommodates uncensored data by evaluating the agreement between predicted and observed orderings of rejection values. The concordance index ranges from 0.5, indicating complete randomness, to 1.0, indicating perfect concordance. For the subset of uncensored data, R^2^, root mean squared error (RMSE), and mean absolute error (MAE) were also calculated to enable comparison with previously published models.

### Model Interpretation

SHapley Additive exPlanations (SHAP) values were used to generate globally ranked feature importance scores and identify pairwise interaction effects, providing insight into both the individual and combined influence of model inputs on PFAS rejection predictions. SHAP values are calculated by computing the marginal contribution of each feature to the model’s prediction, averaged over all possible combinations of feature subsets, following a game theory approach.^23^ To further interpret model behavior, individual conditional expectation (ICE) plots were generated. ICE plots allow for the observation of heterogeneity in model response across data points and help identify how feature effects differ at low versus high rejection values. For each ICE plot, predictions were made by varying the feature of interest while holding all other features constant at randomly sampled values. The average of these predictions was overlaid on the ICE plot as a partial dependence line, representing the average effect of the feature across the distribution of other inputs. To enhance interpretability, all predictions were converted back into the original rejection scale. In addition to single-feature analyses, the model was also used to generate predictions across selected subsets of parameters to explore specific feature interactions.

## Results and Discussion

### Model Accuracy and Feature Importance

A data set comprising 2353 PFAS rejection data points from 60 literature studies on PFAS removal with 111 commercial and lab-synthesized RO and NF membranes was compiled. The most frequently tested commercial membrane was NF270, accounting for 27% of the data set (645 data points), followed by NF90 with 9% (219 data points). Lab-fabricated membranes represented 39% of the data set or 914 data points (Figure 2A). Membrane water permeance ranged from 1.3 to 75 L m^–2^ h^–1^ bar^–1^, with lab-synthesized membranes primarily spanning the higher end of this range. In addition to a diverse set of membranes, the data set includes 44 different PFAS from eight classes. Perfluoroalkyl carboxylic acids (PFCAs) and perfluoroalkyl sulfonic acids (PFSAs) dominate existing literature data, which together make up almost 90% of all data points (Figure 2B). Most studies evaluated performance using PFAS in ultrapure water (35%) or model waters (49%), with only 15% of testing conducted with real waters. Model waters typically contained either salts (ionic strength <20 mM) or organic matter (dissolved organic carbon <5 mg L^–1^) but infrequently both (Figure 2C). Although certain membrane types and PFAS classes are overrepresented, with 111 unique membranes and 44 PFAS, the size and diversity of the data set allow for meaningful evaluation of numerous predictors influencing PFAS rejection.

**Figure 2:**
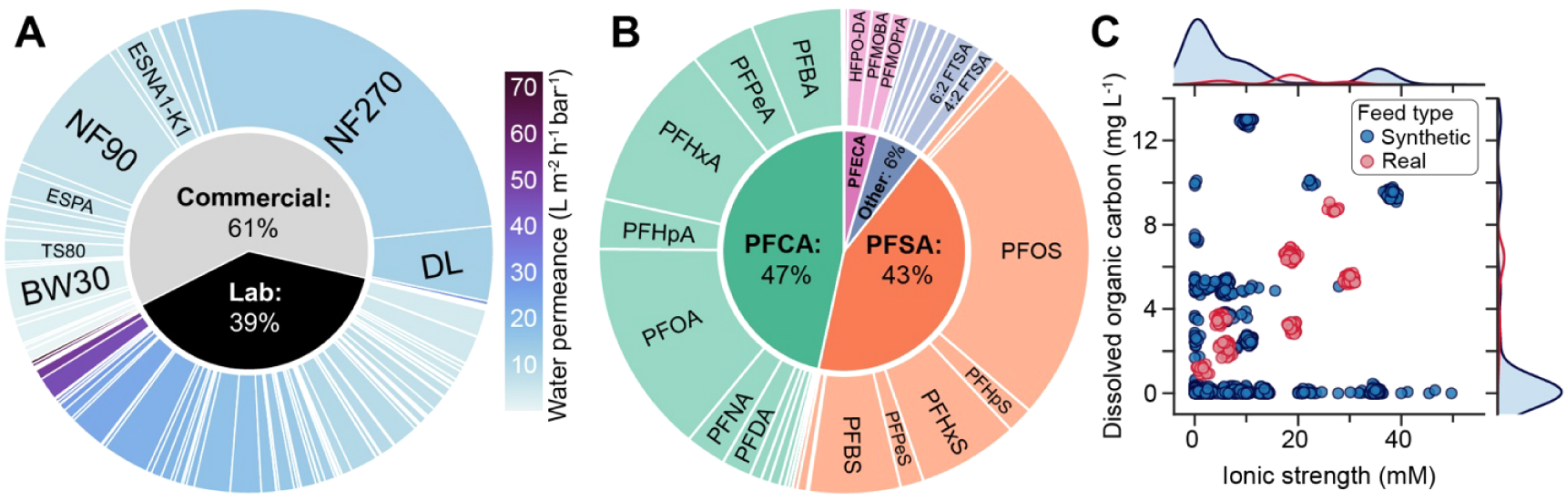
Summary of the literature-derived data set comprising 2353 data points extracted from 60 studies investigating PFAS removal with RO and NF membranes. (A) Distribution of membrane type shaded by membrane pure water permeance. Membranes are separated into commercial and laboratory-synthesized membranes, with the most frequently tested commercial membranes labeled. (B) Distribution of the PFAS classes and compounds in the data set. (C) Feedwater composition, showing dissolved organic carbon concentration and ionic strength for synthetic waters (blue) and real environmental or industrial samples (red). Marginal distributions of ionic strength and dissolved organic carbon concentration are shown along the respective axes. Outliers at 100 and 1000 mM ionic strength are not shown.

To aid in interpreting the diverse literature-derived data set, an XGBoost regression model was trained and tested to predict PFAS rejection and identify key factors governing PFAS rejection in the medium-to-high retention range (*R* > 0.50). The model demonstrated strong predictive accuracy, with close agreement between predicted and observed rejection values (Figure 3A). For the test set, the negative log-likelihood and C-index were 1.91 and 0.89, respectively. Model performance was consistent across the five folds used during cross-validation, indicating stability across diverse experimental conditions tested in literature. To enable comparison with previously published machine learning models for predicting PFAS rejection, RMSE, MAE, and R^2^ were also calculated for uncensored data (rejection values within analytical detection limits). Overall, the model achieves performance comparable to prior machine learning models for predicting organic contaminant rejection in membrane systems (Table 1). Strong test-set results across a diverse data set of PFAS, membranes, and water matrices indicate the model’s capacity to interpolate behavior within the established feature space. Together, these results demonstrate a reliable foundation to enable model interpretation and mechanistic analysis in subsequent sections.

**Table 1:** Model Evaluation Metrics for Cross-Validation and Test Datasets^a^

Reference	Solute class	No. of compounds	Data set size	No. of features	n-loglikelihood	C-index	RMSE (%)	MAE (%)	R^2^
This study (Cross-validation)	PFAS	44	2353	13	1.03 ± 0.10	0.86 ± 0.01	5.36 ± 0.41	2.86 ± 0.21	0.80 ± 0.04
This study (Test)					1.91	0.89	5.80	2.70	0.86
Ref 20	PFAS	21	457	8	-	-	8.4	6.4	-
Ref 19	PFAS	1	289	8	-	-	2.98	-	0.94
Ref 18	PFAS	1	291	8	-	-	2.86	-	0.89
Ref 17	Organic	228	1906	-	-	-	5.25	-	0.88
Ref 24	Organic	81	701	12	-	-	8.85	-	0.96
Ref 25	Organic	276	2102	15	-	-	7.81	6.70	0.87
Ref 26	Organic	19	890	6	-	-	14.61	9.72	0.74

a RMSE, MAE, and R^2^ values are calculated for uncensored data only while n-loglikelihood and concordance index are calculated for the entire dataset. For comparison, evaluation metrics from other studies using machine learning to predict rejection of organic solutes in RO and NF membranes are also shown. RMSE and MAE values are calculated using percent rejection for ease of comparison with other models, whereas other variables are reported based on logit­(*R*) scale.

**Figure 3:**
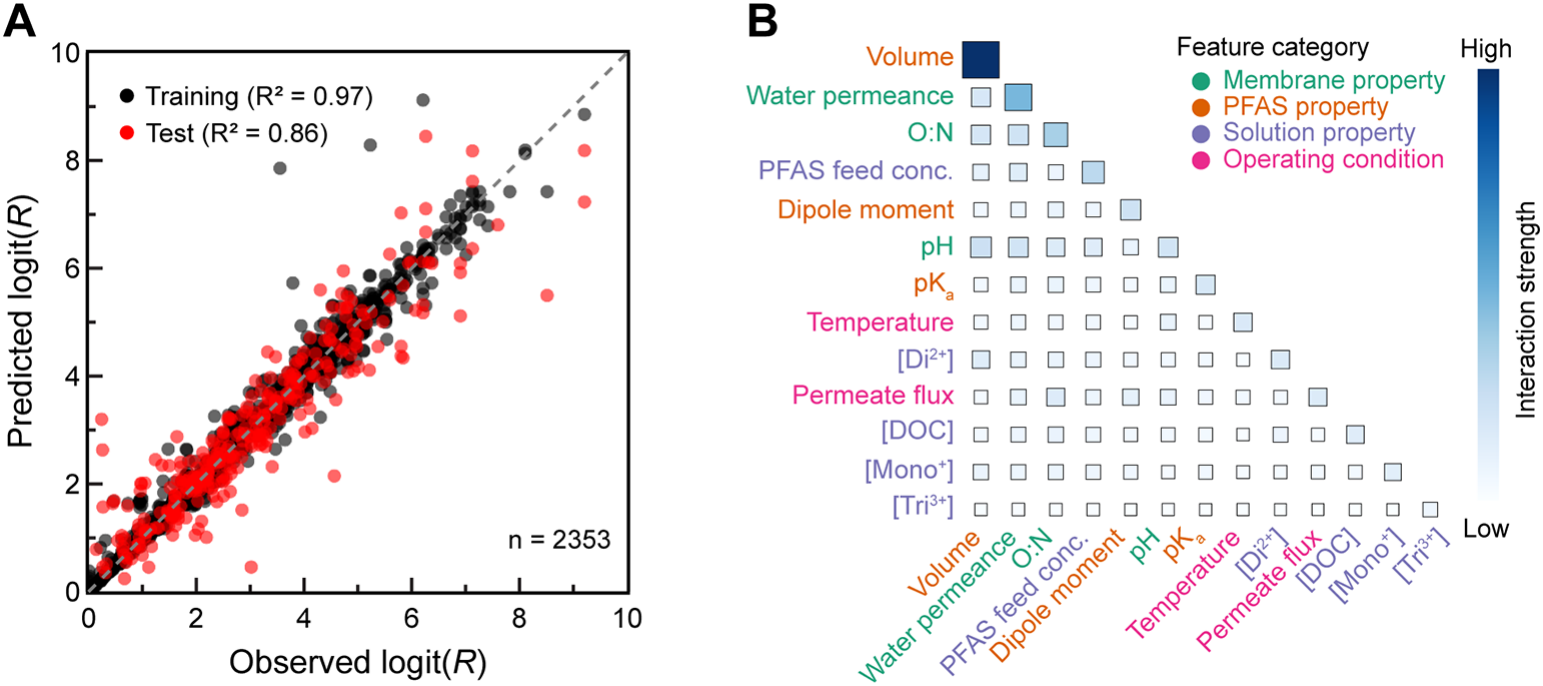
(A) Logit of model-predicted and observed rejection values in the medium-to-high retention range (*R* > 0.50) for uncensored data in training (2000 points) and test (353 points) sets. R^2^ value shown is for uncensored data only. (B) Heatmap of mean absolute SHAP interaction values. Diagonal corresponds to the main effect of a feature while off-diagonal values indicate strength of interaction pair. High interaction strength indicates a stronger effect on rejection.

The relative importance of model inputs and their pairwise interactions is quantified through SHAP analysis (Figure 3B). PFAS volume and membrane water permeance exhibited the strongest influence on rejection, accounting for 42% of the total main effect contribution across the model. The dominance of these two isolated factors suggests that size exclusion is a critical rejection mechanism under the conditions represented. Solution properties (background salt concentrations, organic matter, and PFAS feed concentration) together account for 22% of the total main effect. Notable pairwise interactions were observed between PFAS volume and membrane permeance, O:N ratio, and pH, suggesting that the effect of membrane properties on rejection depends on PFAS size. High interaction strengths indicate that PFAS rejection is governed by coupled effects of membrane, solution, and solute properties, motivating individual conditional expectation (ICE) analyses in the following sections to disentangle their respective contributions.

### Effect of Membrane Properties

To evaluate the influence of membrane properties on PFAS rejection, ICE analysis was used to probe the independent effect of each property on rejection. In this framework, individual curves represent the model’s prediction for a specific set of input parameters as one feature is varied, while the aggregated trend illustrates the partial dependence (average response) across the entire data set. The isolated effect of membrane water permeance was analyzed, and predicted PFAS rejection decreased with increasing membrane water permeance, with the sharpest drop occurring above 20 L m^–2^ h^–1^ bar^–1^ (Figure 4A). While water permeance is a complex function of multiple membrane properties, this significant decrease is consistent with reduced size exclusion from larger membrane void sizes at higher permeabilities, indicating an important contribution of steric effects.^27^ Most tested membranes cluster in the tighter, higher-rejection range, with few data points above 25 L m^–2^ h^–1^ bar^–1^ (Figure 4A, rug plot). Consequently, predictions at permeabilities above this threshold are informed by relatively sparse data. These higher permeance membranes typically fall within the ultrafiltration regime, where void sizes are likely too large for practical PFAS removal.

**Figure 4:**
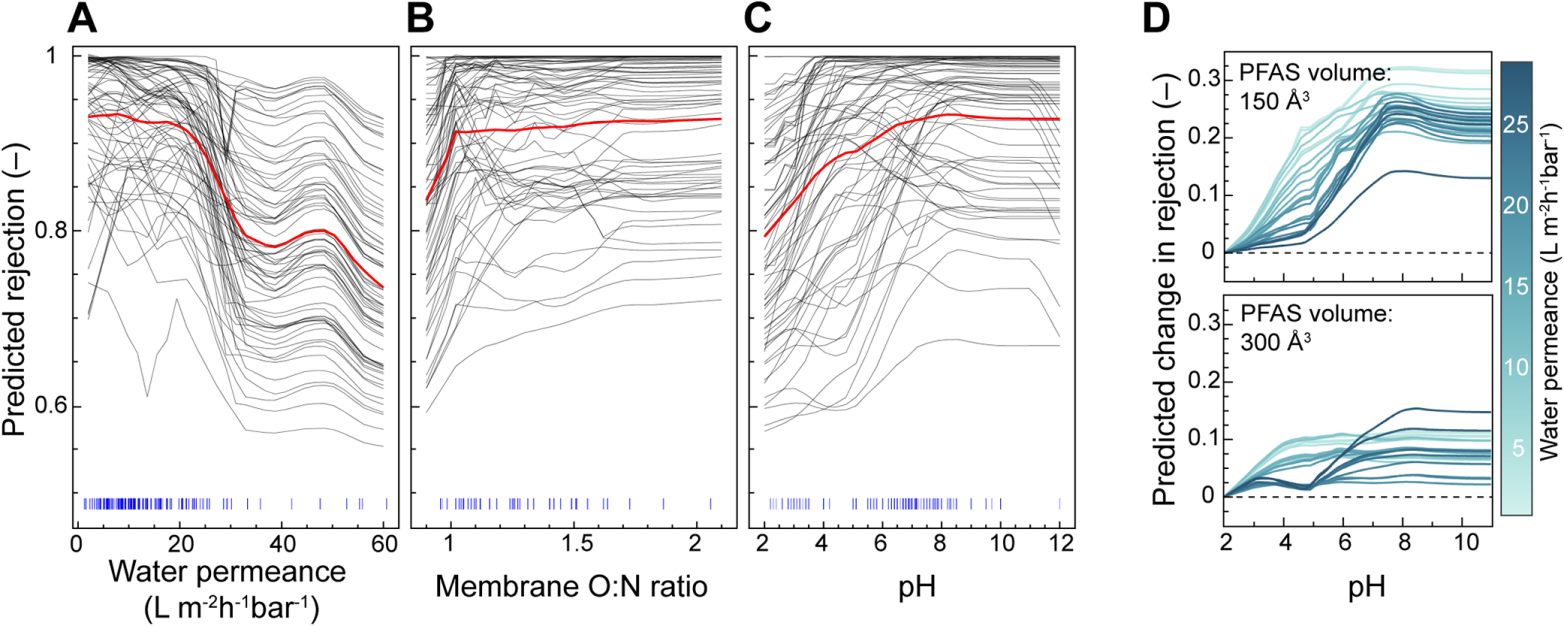
Effect of (A) membrane pure water permeance, (B) O:N ratio, and (C) pH on rejection visualized using ICE plots. Each gray line represents the model response to changes in the respective feature for a randomly sampled set of input parameters while keeping all other features fixed. The red line indicates the average effect (partial dependence) across a random sample of 70 observations, showing the overall trend of how each feature influences the model predictions. Blue tick marks (rug plots) indicate the location and density of feature values in the data set. (D) Predicted rejection in a pure water feed across a range of pH and membrane permeabilities at PFAS molecular volumes of 150 Å^3^ (top) and 300 Å^3^ (bottom).

Variation in predicted PFAS rejection with membrane O:N ratio and feedwater pH reflects the influence of membrane surface charge on PFAS removal. Average predicted rejection increases with increasing O:N ratio, plateauing around a ratio of 2.0 (Figure 4B). Recorded O:N ratios were limited to polyamide-based membranes, where higher ratios indicate a greater fraction of carboxyl groups and higher density of negative surface charge.^28–31^ Although a higher O:N ratio can also indicate lower cross-linking density, the ICE plots decouple these effects by holding water permeance constant. The weak Spearman correlation between O:N and permeance in this data set (ρ = −0.15) indicates minimal collinearity, enabling independent interpretation of their effects (Figure S2). The observed positive trend therefore reflects enhanced electrostatic repulsion of anionic PFAS. The link between O:N ratio and surface charge in governing PFAS removal has been observed in membrane modification studies. For example, Qi et al. demonstrated that incorporating a poly­(vinyl alcohol) interlayer into a polyamide thin film composite membrane increased O:N ratio and decreased membrane zeta potential, increasing perfluorobutanoic acid (PFBA) rejection from 77.3% to 85.6%.^32^ Similarly, Zhang et al. observed that modified metal–organic framework nanosheets increased surface carboxyl group density of a polyamide membrane and enhanced the rejection of 11 PFAS particularly for short-chain species, where electrostatic repulsion plays a more significant role than steric effects.^29^

Predicted PFAS rejection responds strongly to feedwater pH due to pH-dependent changes in membrane charge state. Approximately 80% of the data set comprises polyamide-based membranes, whose surface charge varies with feedwater pH due to ionization of amine and carboxyl groups. At low pH, protonated amine groups impart a positive charge, while carboxylates at higher pH impart a negative charge. Carboxyl groups exhibit two apparent p*K*_a_ values depending on whether they are located on the membrane surface or within the polymer matrix, yielding a membrane isoelectric point typically between pH 3 and 5.^33–35^ Accordingly, predicted PFAS rejection increases with pH, with the steepest rise occurring near pH 3 where polyamide-based membranes transition from neutral to negatively charged (Figure 4C). Because the data set is dominated by anionic PFAS, this increase primarily reflects enhanced electrostatic repulsion. Rejection continues to rise moderately up to about pH 8 before plateauing, likely due to charge saturation. The magnitude of this pH effect depends on both PFAS size and membrane permeance, as indicated by SHAP interaction analysis (Figure 3B). Systems dominated by steric rejection (i.e., lower permeance membrane and larger PFAS) show lower sensitivity to feedwater pH whereas higher-permeance membranes or smaller PFAS display stronger pH-dependent responses that reflect greater contribution of charge-based interactions (Figure 4D). These results align with findings from Ma et al., who reported minimal pH sensitivity from pH 4 to 10 for perfluorooctanoic acid (PFOA) rejection with a tight RO membrane in contrast to greater pH dependence for PFBA and for PFOA with a looser NF membrane.^36^ Overall, trends in PFAS rejection with membrane properties reveal coupled steric and electrostatic mechanisms, with electrostatic contributions becoming more pronounced as steric exclusion weakens.

### Effects of Water Matrix and Operating Conditions

The impact of background ions on PFAS rejection was evaluated using centered-ICE plots to quantify the change in predicted rejection relative to conditions without the ion of interest present (Figure 5; ICE plots without centering are shown in Figure S4). Averaged across randomly sampled experimental conditions, predicted PFAS rejection shows negligible change with monovalent ion concentration (Figure 5A). This trend is consistent with the low main effect strength for monovalent ion concentration (Figure 3B). However, centered-ICE curves reveal heterogeneous responses across factor combinations. Model predictions were therefore generated for controlled subsets of input parameters. For a loose NF membrane, PFAS rejection initially increases with monovalent cation concentration up to approximately 8 mM under acidic conditions and 5 mM under alkaline conditions (Figure 6A). This effect is hypothesized to arise from PFAS-cation complexation, which increases effective solute size and enhances steric exclusion. At low monovalent cation concentrations, prior studies report increased PFAS rejection particularly under acidic conditions where electrostatic repulsion is minimal and steric interactions dominate.^37–40^ This mechanism is supported by Zhao et al., who identified Na-PFOS coordination via UPLC-MS/MS, and Liu et al.’s observations of increased perfluorobutanesulfonic acid (PFBS) particle sizes in the presence of K^+^ and Na^+^.^37,39^ At higher ionic strengths, predicted rejection decreases as charge shielding weakens electrostatic repulsion. This decline is more pronounced for smaller PFAS, which rely more strongly on electrostatic interactions for rejection. Consistent with this behavior, studies report reduced PFAS rejection alongside more positive membrane zeta potentials at elevated NaCl levels, suggesting charge screening as the dominant mechanism driving the observed decrease.^14,36^ Few studies investigated salt concentrations above 20 mM, so model predictions in this regime are less certain. However, available data suggest competing structural effects. At 1000 mM NaCl, Ma et al. hypothesized that pore swelling reduced size exclusion, whereas Wang et al. reported a reduction in pore size at 100 mM that improved PFOS rejection.^14,36^ Overall, the model suggests a highly nonmonotonic effect of monovalent salts on PFAS rejection in which competing mechanisms, including complexation, charge shielding, and pore structure changes, may offset one another. The net effect ultimately depends on PFAS size, ionic strength, and membrane properties.

**Figure 5:**
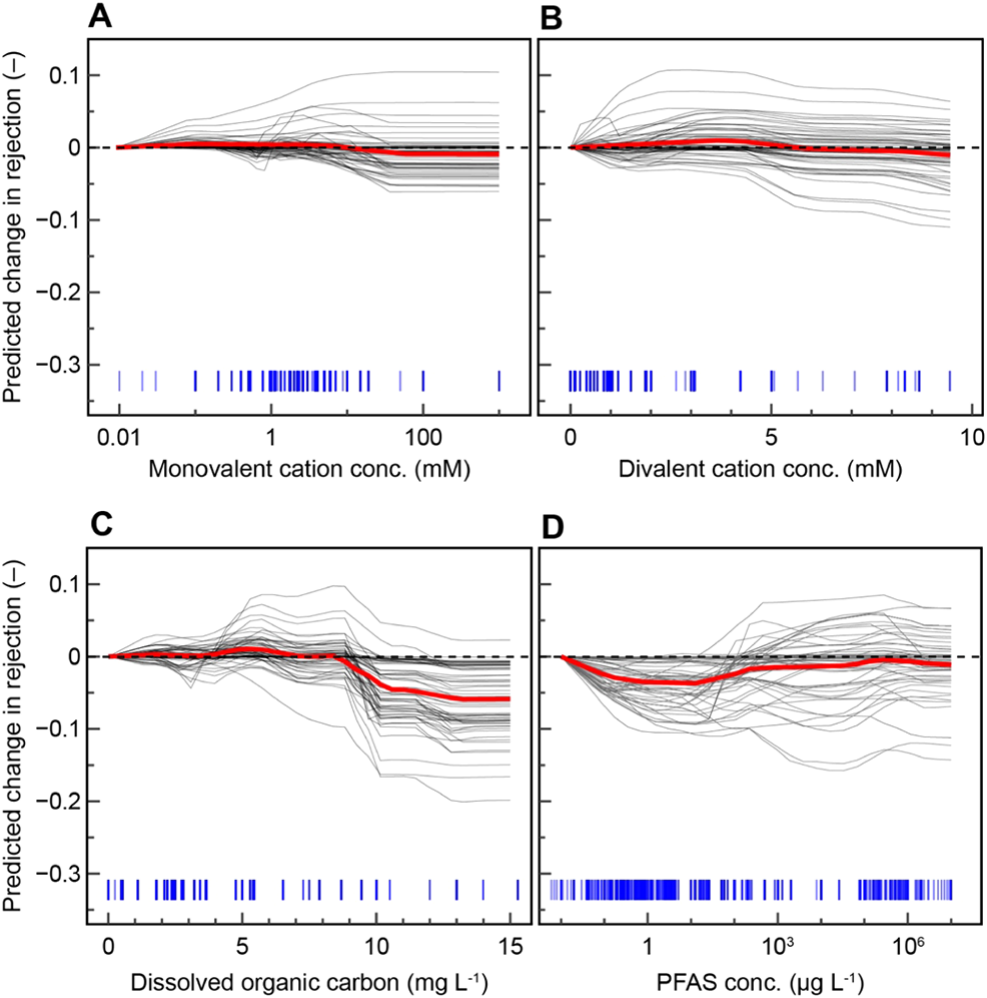
Effect of (A) monovalent cation concentration, (B) divalent cation concentration, (C) DOC concentration, and (D) initial PFAS feed concentration on rejection visualized using centered ICE plots. Each gray line represents the model response to changes in the respective feature for a randomly sampled set of input parameters while keeping all other features fixed. The red line indicates the average effect (partial dependence) across a random sample of 70 observations, showing the overall trend of how each feature influences the model’s predictions. Blue tick marks indicate the distribution of training data values for each feature.

**Figure 6:**
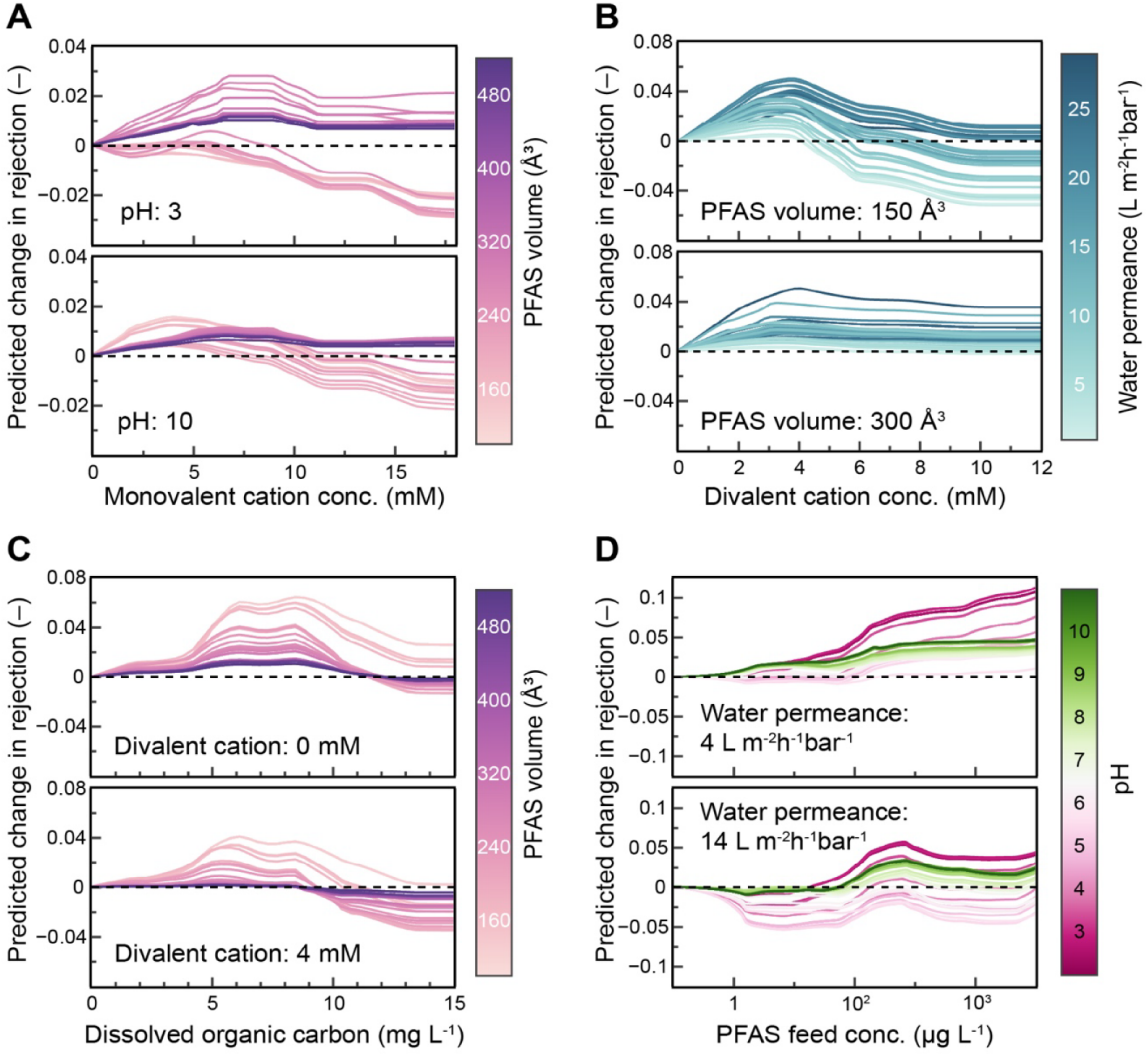
Predicted change in rejection as a function of the following conditions: (A) monovalent cation concentration across different PFAS molecular volumes at pH 3 and 10 with a membrane water permeance of 12 L m^–2^ h^–1^ bar^–1^; (B) divalent cation concentration with different membrane permeabilities for PFAS volumes of 150 and 300 Å^3^; (C) DOC across different PFAS molecular volumes in pure water and at divalent cation concentration of 4 mM for a membrane water permeance of 12 L m^–2^ h^–1^ bar^–1^; (D) PFAS feed concentration in pure water for PFAS with a molecular volume of 250 Å^3^. Parameters not specified are fixed at the mean of the data set.

Divalent cations produce a larger initial increase in predicted PFAS rejection at low concentrations compared to monovalent cations (Figure 5B). This response can arise from complexation and intermolecular bridging, which increase apparent particle size. Evidence for these mechanisms has been reported across multiple studies. Zhao et al. identified PFOS-cation-PFOS complexes via UPLC-MS/MS and confirmed coordination with two PFOS molecules with X-ray photoelectron spectroscopy and density functional theory analysis.^37^ Similarly, Liu et al. measured an increase in particle size in PFBS stock solutions containing Ca^2+^ and Mg^2+^.^38^ In addition to increasing effective solute size, studies report that divalent cations promote PFAS adsorption to the membrane surface, leading to partial pore blockage that further improves rejection. These mechanisms are supported by experimental evidence of reduced permeate flux and the formation of thicker fouling layers in the presence of divalent cations.^37,41–43^ Model predictions further show that for smaller PFAS, rejection begins to decline once divalent salt concentrations exceed roughly 4 mM (Figure 6B). This behavior likely indicates a transition where reduced electrostatic repulsion from charge shielding starts to outweigh the benefits of ion bridging. Liu et al. reported reduced magnitude of the membrane’s negative zeta potential in the presence of PFBS and PFOA with Ca^2+^ and Mg^2+^ at concentrations up to 3 mM.^38,39^ Evidence of the resulting performance decline is also observed in Soriano et al., who found reduced perfluorohexanoic acid rejection when testing solutions containing monovalent and divalent salts with total ionic strengths between 13 and 45 mM.^44^

For trivalent salts, the model predicts an overall decrease in PFAS rejection with increasing concentration (Figure S4C). Liu et al. hypothesized that hydrolysis of Fe^3+^ forms large aggregates with PFBS and Fe­(OH)_3_, yielding a fouling layer that promotes cake-enhanced concentration polarization and reduced rejection.^38^ In contrast, Zhao et al. found that complexation with Fe^3+^ enhanced PFOS rejection by strengthening size exclusion.^37^ However, membrane performance and characterization data with trivalent salts are sparse and limited to a few PFAS types, leaving interactions and mechanisms uncertain. Additional systematic testing across a broader range of PFAS and membranes would be needed to clarify how trivalent ions influence rejection behavior.

Predicted PFAS rejection also varies nonmonotonically with DOC concentration. At low DOC levels, predicted rejection increases with DOC, with changes leveling off near 6 mg L^–1^ on average across all input conditions (Figure 5C). In this concentration range, studies report that organic matter adsorbs to the membrane surface and increases membrane negative surface charge, leading to partial pore blockage and stronger electrostatic repulsion. For example, Liu et al. observed more negative membrane surface charge, formation of a loose cake layer, and declining permeate flux in feeds containing PFOA with sodium alginate or humic acid.^38^ Similarly, Wang et al. found that sodium alginate decreased membrane surface potential and enhanced adsorption of PFOS and PFBS.^14^ Complexation between PFAS and organic matter may also increase apparent PFAS size, further strengthening size exclusion.^39^ Beyond 6 mg L^–1^, the model predicts a decrease in rejection. At these higher DOC levels, fouling layers can induce cake-enhanced concentration polarization and hinder rejection. Steinle-Darling reported lower perfluorooctanesulfonamide and 6:2 fluorotelomer sulfonic acid rejection for sodium-alginate-fouled membranes, while Liu et al. observed compact fouling layers reduced PFAS rejection, especially for short-chain species.^45,46^ The combined presence of organic matter and inorganic ions further influences rejection behavior, and there is a greater decrease in predicted rejection with DOC in the presence of 2 mM divalent cations than with no added salts (Figure 6C). The coexistence of divalent cations and organic matter can promote the formation of loose fouling layers that exacerbate concentration polarization.^14,43,47,48^ These interactions highlight the importance of evaluating PFAS rejection under realistic water compositions where salts and organic matter coexist and jointly influence membrane performance. Although low concentrations of DOC or salts alone may improve rejection, many studies report lower rejection in real water samples compared to deionized water.^49,50^ Effects from fouling layer formation, complexation, and changes in membrane surface charge ultimately depend on DOC concentration, PFAS size, and coexisting inorganic ions.

Model predictions indicate that the effect of initial PFAS feed concentration on rejection depends on membrane adsorption capacity and solution pH. On average, predicted rejection decreases with initial PFAS concentration up to approximately 50 μg L^–1^ (Figure 5D). In this lower concentration range, increasing PFAS accumulation may increase concentration polarization effects.^45,51,52^ This behavior is consistent with studies reporting lower rejection at higher recovery ratios.^50,53^ At higher feed concentrations, however, significant adsorption may cause partial pore blockage, enhancing steric exclusion.^14,52,54^ Additionally, self-aggregation of PFAS at concentrations as low as 0.001 times the critical micelle concentration may further enhance size exclusion at mg L^–1^ levels.^53,55,56^ These mechanisms shift with membrane permeance and feed pH (Figure 6D). The magnitude of concentration-dependent effects on rejection is greatest under acidic conditions, where weaker electrostatic repulsion promotes adsorption.^57,58^ In contrast, tighter membranes exhibit a stronger net improvement in rejection at higher PFAS concentrations, as adsorption-induced pore narrowing helps counteract concentration polarization losses. Given this sensitivity to PFAS feed concentration, interpreting results from experimental studies requires careful consideration of testing conditions. Most PFAS testing in the literature is conducted at concentrations far above environmentally relevant levels of ng L^–1^ to low μg L^–1^ (Figure 5D, rug plot). At these trace levels, the relative importance of rejection mechanisms may shift; physical phenomena, such as adsorption or pore blockage, documented in bench-scale studies, may not be representative of rejection behavior at low PFAS concentrations. Future work should therefore aim to establish transfer functions to extrapolate membrane performance to realistic PFAS concentrations.

PFAS rejection is also affected by operating conditions, including temperature and permeate flux. Although most experimental data are concentrated near room temperature, limiting confidence outside this range, model predictions suggest rejection remains constant from 5 to 20 °C before declining from 20 to 35 °C (Figure S5). The magnitude of this effect varies by membrane: while Wu and Boonya-Atichart et al. observed negligible temperature sensitivity, Chen et al. and Hang et al. reported measurable differences in rejection with lab synthesized and commercial polyamide membranes due to thermal expansion of the membrane voids.^59–62^ Predicted rejection gradually declines with increasing permeate flux, which depends on both membrane permeance and applied pressure (Figure S5). Because only observed (rather than intrinsic) rejection values were modeled, this decline likely reflects concentration polarization effects at higher fluxes. While related, the trend with membrane permeance exhibits a sharper, continuous decrease in rejection, indicating the physical link between permeance and steric exclusion. Other operational variables, such as filtration time and percent recovery, were not explicitly included in the model due to limited data availability. However, several studies report notable declines in PFAS rejection during extended filtration, particularly in the presence of organic foulants.^14,60^ Incorporating such time-dependent operational factors into future analyses could improve understanding of process performance under realistic treatment scenarios.

### Effect of PFAS Properties

Descriptors for PFAS geometry and charge were evaluated as candidate model features to identify and characterize how PFAS physicochemical properties influence rejection. Because most PFAS investigated are assumed to have a linear fluorocarbon backbone, many geometric descriptors are highly correlated. Thus, a single representative size metric was selected to avoid redundancy. Spearman correlation coefficients were calculated between PFAS rejection and several size-related parameters, including molecular weight, volume, minimum and maximum projection area and radius, to determine the most informative descriptor (Figure S3). Among these, PFAS volume showed the strongest correlation with rejection and was therefore selected as a model feature. As expected, predicted rejection increased with molecular volume (Figure S6A). Beyond this main effect, PFAS volume also interacted with membrane and feedwater conditions to influence the dominant transport mechanisms, as discussed in previous sections. The distribution of experimental testing to date is heavily skewed toward PFAS with volumes greater than 180 Å^3^ or carbon chain lengths greater than 6 (Figure S6A, rug plot), whereas ultrashort-chain PFAS, such as trifluoroacetic acid, account for only 1% of observations. Expanding representation of these smaller species is critical, as they are increasingly prevalent as degradation byproducts and replacements for phased-out long-chain species. Additionally, their small size and high mobility may pose greater challenges for membrane-based removal and therefore warrant further investigation.^12,63^

Differences in PFAS functional group acidity influence membrane-solute electrostatic interactions, contributing to variability in rejection behavior among similar molecular sizes. For PFAS with p*K*_a_ values above 3, predicted rejection decreases (Figure S6B). In acidic feedwaters, these species are less dissociated, weakening electrostatic repulsion with negatively charged membranes. Accordingly, neutral species such as perfluoroalkane sulfonamides (FASAs) exhibit lower rejection than similarly sized anionic PFAS.^64,65^ Some studies report higher rejection of PFCAs than PFSAs of equal carbon chain length, attributing this behavior to the stronger partial negative charge on the carboxylate group.^53,66^ However, others observe higher PFSA rejection or no clear pattern across chain lengths.^11,46,57,62^ Comparisons of rejection behavior across PFAS classes in studies using natural waters are further complicated by differences in feed concentration and matrix composition. To reconcile these mixed findings, the model was used to predict rejection for different PFAS species at pH 3 and 8 while holding other variables constant (Figure S7). Model predictions indicate higher PFCA rejection than PFSA at similar molecular volumes, with differences becoming more pronounced under alkaline conditions. Predicted rejection of perfluoroalkyl ether acids is comparable to that of PFCAs, following similar trends with molecular volume and feed pH, while fluorotelomer sulfonic acids exhibit a sharper size-dependent increase in predicted rejection. Rejection behavior among these anionic PFAS classes appear strongly system-dependent, and existing evidence does not support a universal conclusion that any class is consistently rejected more than another at the same chain length.

Additional physicochemical properties reported to influence organic micropollutant rejection in NF and RO systems, including molecular polarity and hydrophobicity, were evaluated. Predicted PFAS rejection decreases with increasing molecular polarity, parametrized by dipole moment (Figure S6C). A larger dipole moment may enable molecules to orient in a way that minimizes electrostatic repulsion at the membrane interface and thereby facilitates permeation.^67^ Hydrophobicity, characterized by logK_ow_, has also been identified as an important predictor for organic contaminants in NF and RO systems, where higher logK_ow_ correlates with stronger sorption and reduced permeation.^67,68,69^ However, because logK_ow_ is strongly correlated with PFAS size, it was excluded from the model. Ultimately, molecular size and charge state remain the dominant determinants of PFAS rejection while other molecular properties contribute comparatively minor effects.

### Potential for Emerging Membranes

Recent advancements in membrane materials to overcome permeability-selectivity trade-off for PFAS highlight promising design strategies. Methods targeting high hydrophilicity and negative surface charge achieve high permeability and rejection of long-chain PFAS (C > 6), extending performance beyond the range of most commercial membranes, which remain clustered at lower permeabilities (Figure 7A). To achieve these improvements in membrane performance, studies have employed surface modification and interfacial engineering approaches, such as nanomaterial-enhanced interlayers and functional coatings. For example, Ma J. et al. incorporated MXene nanosheets to enhance membrane hydrophilicity, reducing water contact angle by 27% and increasing permeance from 9 to 12 L m^–2^ h^–1^ bar^–1^ while maintaining comparable rejection.^70^ Similarly, Luo et al. demonstrated that incorporation of organically modified montmorillonite improved membrane hydrophilicity, resulting in higher water permeance and PFOS rejection.^71^ Other studies have focused on increasing membrane negative surface charge to strengthen electrostatic repulsion of anionic PFAS. Le et al. embedded MXene nanosheets to increase both hydrophilicity and negative surface charge, achieving PFOS rejection of 96% while doubling water permeance relative to the control.^72^ Abdikheibari et al. used BN­(NH_2_) nanosheets to improve wetting and surface charge, reporting increases in both permeance and rejection.^73^ Additional approaches include polyelectrolyte multilayers and metal–organic frameworks (MOFs) to enhance negative surface charge and improve PFAS rejection via electrostatic repulsion.^29,74–77^ However, lab-synthesized membranes are rarely tested or optimized for ultrashort (C ≤ 3) or short- chain (C ≤ 6) PFAS (Figure 7A), and addressing performance for these species remains a critical area for future membrane development.

**Figure 7:**
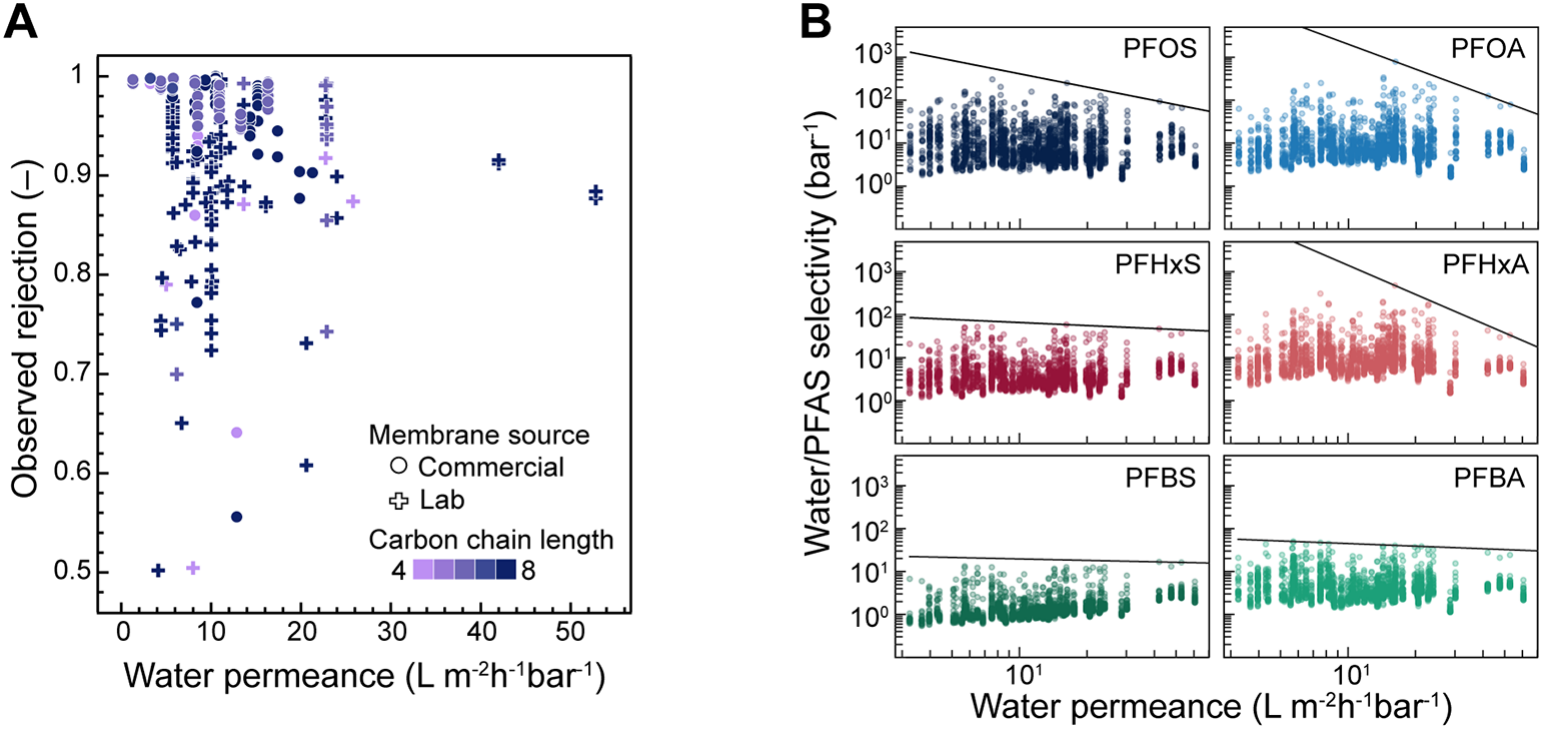
(A) Observed PFAS rejection in pure water feeds with neutral or alkaline pH versus membrane water permeance. Only PFAS for which experiments were conducted with both lab-synthesized (crosses) and commercial membranes (circles) are shown. Data points are colored by PFAS carbon chain length. (B) Simulated PFAS selectivity versus membrane water permeance and empirically fit upper bound for water/PFAS separation.

To enable systematic comparison of membrane performance and PFAS chemistries across studies, the model was applied to estimate permeability-selectivity trade-offs for different membranes in the data set. Because intrinsic rejection and concentration polarization moduli were rarely reported, PFAS rejection in pure water feeds was simulated for reported membrane properties across a range of initial PFAS feed concentrations and fluxes to capture experimental variability. These simulations enable evaluation of membrane performance across a broader set of PFAS chemistries than were explicitly tested for each membrane, providing a more complete mapping of the design space. From these predictions, an empirical upper bound of selectivity was constructed based on a quantile regression (τ = 0.95) of the running maximum (Figure 7B). For C6 and C8 PFAS, membranes incorporating MOFs developed by Zhang et al. and Bi et al. perform near this empirical frontier. In Zhang et al., MOF incorporation produced a denser membrane structure with a higher fraction of carboxyl groups, which enhanced electrostatic repulsion.^29^ Bi et al. similarly reported improved water flux and PFAS retention due to increased hydrogen bonding and favorable electrostatic interactions.^78^ For PFBA and PFBS, membranes synthesized by Qi et al. and Chen et al. exhibit high performance near this empirical bound. These membranes incorporated poly­(vinyl alcohol) and hyaluronic acid interlayers, respectively, which enhanced hydrophilicity, increased cross-linking density, and yielded more negatively charged surfaces that were advantageous for improving short-chain PFAS rejection.^32,62^ Overall, the performance benchmarks defined here help contextualize the progress of emerging membrane materials and guide future efforts for enhancing PFAS removal.

## Implications

This meta-analysis synthesizes PFAS rejection data across studies with differing testing conditions, including flux, feed concentration, and temperature, enabling a comprehensive understanding of PFAS rejection trends. SHAP analyses reveal membrane permeance and PFAS molecular size as dominant main-effect predictors of PFAS rejection, underscoring the importance of steric exclusion. When interaction effects are considered, parameters governing membrane charge, particularly feedwater pH and membrane O:N ratio, become increasingly influential, especially for small PFAS and loose NF membranes where electrostatic interactions more strongly contribute to rejection. Model predictions further indicate that low concentrations of salts as well as organic matter can improve rejection through complexation and adsorption-driven size exclusion. However, high ionic strength and DOC reduce rejection through charge screening and cake-enhanced concentration polarization, respectively. On average, ionic strength above 8 mM and DOC exceeding 10 mg L^–1^ produces the largest declines in rejection, though the concentration thresholds and magnitude of these effects on rejection depend strongly on PFAS size, membrane properties, and co-occurring feed constituents.

Based on the PFAS rejection trends identified, future membrane development should prioritize strategies that balance high permeability with robust charge-based selectivity, particularly for short-chain PFAS and complex waters where charge screening and fouling are expected to pose the greatest challenges. This study provides permeability-selectivity trade-offs that can be used to evaluate the relative performance of new membranes for PFAS rejection.

Despite the strengths of this analysis, our ability to predict and understand PFAS rejection is inherently limited by the scope of available data, particularly in underexplored regions of the feature space. Most existing PFAS studies examining feed matrix effects vary only single components, with little testing conducted in natural waters or simultaneously varying both salt and organic matter concentrations. Consequently, while our model captures trends within the sampled feature space, limited multicomponent experimental data restricts our ability to fully resolve nonlinear, higher-order interactions typical of complex real-world feeds. Experimental testing has also been heavily concentrated on anionic PFAS, particularly species with chain lengths greater than six. Ultrashort-chain and neutral PFAS remain severely underrepresented. These knowledge gaps are especially critical for more nuanced trends, such as the effect of background ions, where both PFAS properties and membrane characteristics interact to determine the balance of competing mechanisms. To support progress in this area, the complete data set is provided in the Supporting Information, enabling future studies to build upon this work. Continued expansion of testing across a broader range of PFAS chemistries, membrane types, and feedwater conditions will be essential as knowledge of PFAS health risks grows. By consolidating diverse experimental data and applying interpretable machine learning tools to resolve complex PFAS transport interactions, this work offers a foundation for more targeted development of future PFAS remediation strategies.

## Supplementary Material





## Notes

Statistical summary of variables and PFAS included in data set, reported rejection distribution, Spearman correlation among features, and additional visualizations of model predictions (PDF)
Source data (XLSX)
