# Supplementary material for: Mechanistic Insights into PFAS Rejection in Nanofiltration and Reverse Osmosis from Data-Driven Analysis
Source: Environ Sci Technol. 2026 Apr 30;60(18):13711–23. doi: 10.1021/acs.est.6c02287 (PMC13173650; doi:10.1021/acs.est.6c02287)
Supplement: Supplementary file 1 [file es6c02287_si_001.pdf]

Supporting Information for “Mechanistic Insights into PFAS Rejection in Nanofiltration and Reverse Osmosis from Data-Driven Analysis”

Environmental Science & Technology

Yukai Tomsovic[1,2], Siwei Gu[3], Kyle Doudrick[3], and Anthony P. Straub[1,2,4*]

[1]*Department of Mechanical and Process Engineering, ETH Zürich, Zürich 8092, Switzerland*

[2]*Materials Science and Engineering Program, University of Colorado Boulder, Boulder, CO 80309-0428, USA*

[3]*Department of Civil and Environmental Engineering and Earth Sciences, University of Notre Dame, Notre Dame, IN 46556, USA*

[4]*Department of Civil, Environmental and Architectural Engineering, University of Colorado Boulder, Boulder, CO 80309-0428, USA*

Summary: 12 pages, 7 figures, 2 tables

* Corresponding author, E-mail: astraub@ethz.ch

List of Tables and Figures

Table S1. Statistical summary of all quantitative variables collected from literature and fraction of missing values.

Table S2. List of PFAS classes and species included in dataset.

Figure S1. Distribution of rejection values in training and test datasets before (a) and after (b) applying a logit transformation. (c) Logit of rejection versus datapoints in original rejection scale.

Figure S2. Spearman rank correlation coefficient matrix for input features.

Figure S3. Spearman correlation coefficients between logit(*R*) and different PFAS physicochemical properties.

Figure S4. ICE plots for the effect of (a) monovalent cation, (b) divalent cation, (c) trivalent cation, (d) DOC, and (e) initial PFAS feed concentrations.

Figure S5. ICE plots for the effect of (a) operating temperature and (b) permeate flux.

Figure S6. ICE plots for the effect of PFAS (a) molecular volume, (b), pK_a, and (c) dipole moment.

Figure S7. Predicted rejection versus PFAS molecular volume by PFAS class.

Table S1. Statistical summary of all quantitative variables collected from literature and fraction of missing values.

Feature	*Min*	*0.25*	*Median*	*Mean*	*0.75*	*Max*	*Std. Dev.*	*Missing frac.*
Membrane property								
Pure water permeability ($L\ m^{-2}\ h^{-1}\ bar^{-1}$)	1.30	8.20	12.70	13.35	15.13	74.73	9.06	0
Oxygen: nitrogen ratio	0.96	1.16	1.31	1.80	1.31	9.35	1.74	0.28
Feed pH	2.20	6.50	7.00	6.67	7.40	12.00	1.34	0.13
PFAS property								
Molecular weight ($g\ mol^{-1}$)	114.02	314.05	414.06	399.45	500.13	664.02	103.05	0
$logK_{ow}$	-1.04	3.11	4.21	3.85	4.84	7.62	1.20	0
Van der Waals volume ($Å^3$)	70.82	179.33	233.54	228.38	277.54	505.34	61.09	0
Topological polar surface area ($Å^2$)	29.10	37.30	46.53	46.06	54.37	69.23	8.75	0
pK_a	-3.93	-3.32	0.29	-1.29	0.32	3.55	1.90	0
Partial charge	-0.52	-0.52	-0.52	-0.42	-0.31	-0.25	0.10	0
Polarizability ($Å^3$)	5.11	12.76	16.60	16.88	20.82	35.77	4.67	0
Dipole moment (debye)	1.50	1.68	1.78	2.28	1.89	38.00	3.66	0
Minimum projection area ($Å^2$)	19.11	31.30	32.97	33.73	36.44	57.03	5.09	0
Maximum projection area ($Å^2$)	26.60	52.15	64.74	65.46	77.43	151.74	15.90	0
Minimum projection radius (Å)	2.68	3.41	3.55	3.69	3.97	5.41	0.37	0
Maximum projection radius (Å)	3.52	5.26	6.15	6.36	7.44	13.90	1.29	0
Carbon chain length	1	5	8	6.61	8	15	2.08	0
Solution property								
Initial feed concentration ($\mu g\ L^{-1}$)	0.0010	1	100	99708	100	10000000	831618	0.00
Monovalent cation concentration (mM)	0	0	0.77	5.18	4.94	1000	42.36	0.04
Divalent cation concentration (mM)	0	0	0.10	1.81	1.89	9.45	2.76	0.03
Trivalent cation concentration (mM)	0	0	0.00	0.01	0.00	6.00	0.15	0.03
Dissolved organic carbon conc. ($mg\ L^{-1}$)	0	0	0.0	1.93	2.50	30.35	3.64	0.02
Operating condition								
Temperature (°C)	5.00	20.00	25.00	23.38	25.00	36.00	3.34	0.17
Permeate flux ($L\ m^{-2}\ h^{-1}$)	3.55	24.73	48.15	58.82	75.65	307.09	46.12	0.60

Table S2. List of PFAS classes and species included in dataset.

Name	*Abbreviation*	*CAS ID*	*Molecular formula*	*Class*
Trifluoroacetic acid	TFA	76-05-1	C2HF3O2	PFCA
Perfluoropropionic acid	PFPrA	422-64-0	C3HF5O2	PFCA
Perfluorobutanoic acid	PFBA	375-22-4	C4HF7O2	PFCA
Perfluoropentanoic acid	PFPeA	2706-90-3	C5HF9O2	PFCA
Perfluorohexanoic acid	PFHxA	307-24-4	C6HF11O2	PFCA
Perfluoroheptanoic acid	PFHpA	375-85-9	C7HF13O2	PFCA
Perfluorooctanoic acid	PFOA	335-67-1	C8HF15O2	PFCA
Perfluorononanoic acid	PFNA	375-95-1	C9HF17O2	PFCA
Perfluorodecanoic acid	PFDA	335-76-2	C10HF19O2	PFCA
Perfluoroundecanoic acid	PFUnDA	2058-94-8	C11HF21O2	PFCA
Perfluorododecanoic acid	PFDoDA	307-55-1	C12HF23O2	PFCA
Perfluorotridecanoic acid	PFTrDA	72629-94-8	C13HF25O2	PFCA
Perfluorotetradecanoic acid	PFTA	0376-06-07	C14HF27O2	PFCA
Perfluorohexadecanoic acid	PFHxDA	67905-19-5	C16HF31O2	PFCA
Perfluorooctadecanoic acid	PFODA	16517-11-06	C17F35COOH	PFCA
Trifluoromethanesulfonic acid	TFMS	1493-13-6	CHF3O3S	PFSA
Perfluoropropanesulfonic acid	PFPrS	423-41-6	C3HF7O3S	PFSA
Perfluorobutanesulfonic acid	PFBS	375-73-5	C4HF9O3S	PFSA
Perfluoropentanesulfonic acid	PFPeS	2706-91-4	C5HF11O3S	PFSA
Perfluorohexanesulfonic acid	PFHxS	355-46-4	C6HF13O3S	PFSA
Perfluoroheptanesulfonic acid	PFHpS	375-92-8	C7HF15O3S	PFSA
Perfluorooctanesulfonic acid	PFOS	1763-23-1	C8HF17O3S	PFSA
Perfluorononanesulfonic acid	PFNS	68259-12-1	C9HF19SO3	PFSA
Perfluorodecanesulfonic acid	PFDS	335-77-3	C10HF21SO3	PFSA
Perfluoro-3-methoxypropanoic acid	PFMOPrA	377-73-1	C4HF7O3	PFECA
Perfluoro-4-methoxybutanoic acid	PFMOBA	863090-89-5	C5HF9O3	PFECA
Hexafluoropropylene oxide-dimer acid	HFPO-DA	13252-13-6	C6HF11O3	PFECA
4,8-Dioxa-3H-perfluorononanoic acid	DONA	919005-14-4	C7H2F12O4	PFECA
Hexafluoropropylene oxide trimer acid	HFPO-TrA	13252-14-7	C9F17O4H	PFECA
Hexafluoropropylene oxide tetramer acid	HFPO-TeA	65294-16-8	C12HF23O5	PFECA
Perfluoro(4-ethylcyclohexane)sulfonic acid	PFECHS	646-83-3	C8HF15O3S	PFESA
4:2 Fluorotelomer sulfonic acid	4:2 FTSA	757124-72-4	C6H5F9O3S	FTSA
6:2 Fluorotelomer sulfonic acid	6:2 FTSA	27619-97-2	C8H5F13O3S	FTSA
8:2 Fluorotelomer sulfonic acid	8:2 FTSA	39108-34-4	C10H5F17O3S	FTSA
Perfluorooctanesulfonamide	FOSA	754-91-6	C8H2F17NO2S	FASA
N-[3-(dimethylamino)propyl] 1,1,2,2,3,3,4,4,4-nonafluorobutane-1-sulfonamide	PFBSAm	68555-77-1	C9H13F9N2O2S	FASA
6:2 Chlorinated perfluoroether sulfonic acid	6:2 Cl-PFESA	756426-58-1	C8HClF16O4S	Cl-PFESA
8:2 Chlorinated perfluoroether sulfonic acid	8:2 Cl-PFESA	763051-92-9	ClC10F20OSO3H	Cl-PFESA

Name	Abbreviation	CAS ID	Molecular formula	Class
Bis(perfluorohexyl)phosphinic acid	6:6 PFPiA	40143-77-9	C12F24O2P	PFPIA
Perfluorohexylperfluorooctylphosphinic acid	6:8 PFPiA	610800-34-5	C14F30O2HP	PFPIA
Perfluorooctaneylamimopropyl trimethyl ammonium iodide	C1007311I	335-90-0	C14H16F15IN2O	Precursor
2-[dimethyl-[3(2,2,3,3,4,4,5,5,6,6,7,7,8,8,8-pentadecafluorooctanoylamino) propyl] azaniumyl] acetate	T100731	90179-39-8	C15H15F15N2O3	Precursor
Sodium perfluoro nonyloxy benzenesulfonate	OBS	77061-68-8	C15F23NaO4S	Precursor

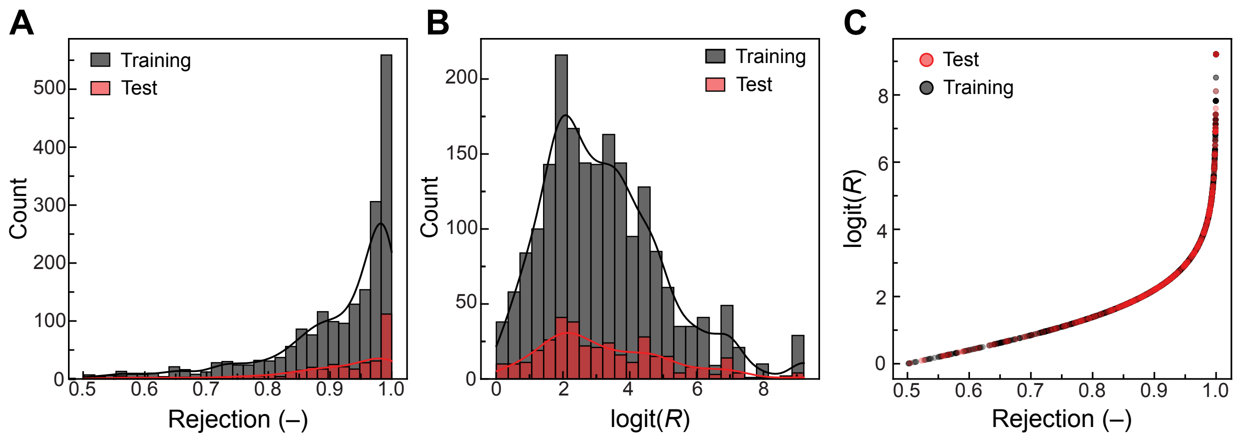

Figure S1. Distribution of rejection values in training and test datasets before (a) and after (b) applying a logit transformation. (c) Logit of rejection versus datapoints in original rejection scale.

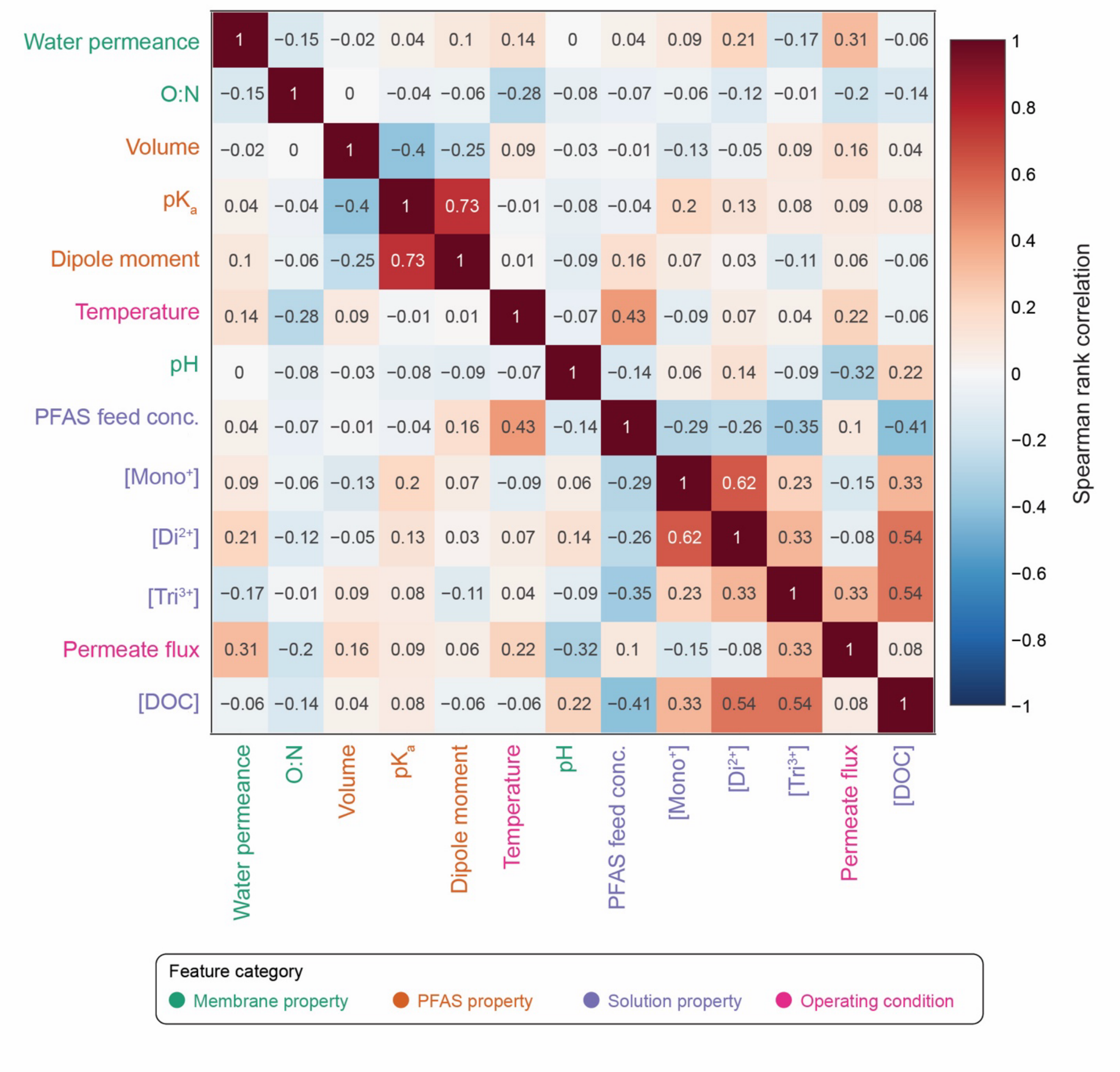

Figure S2. Spearman rank correlation coefficient matrix for input features.

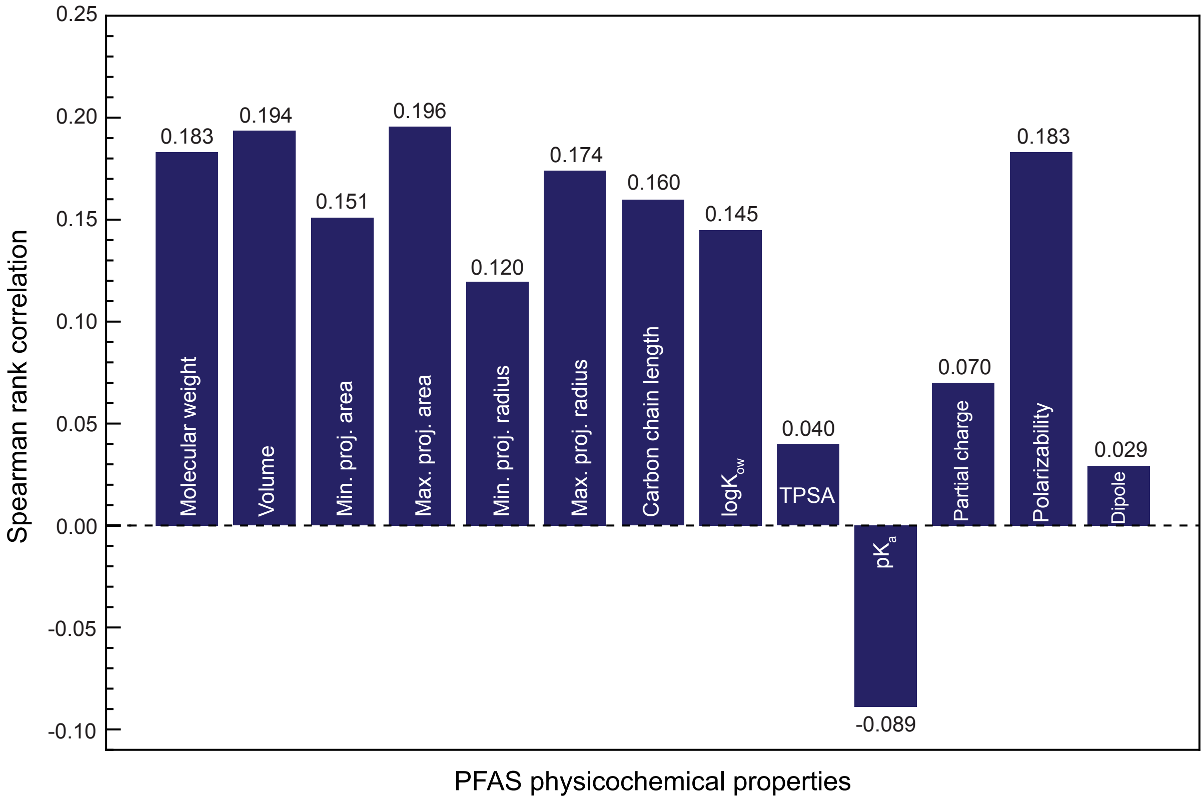

Figure S3. Spearman correlation coefficients between logit(R) and different PFAS physicochemical properties.

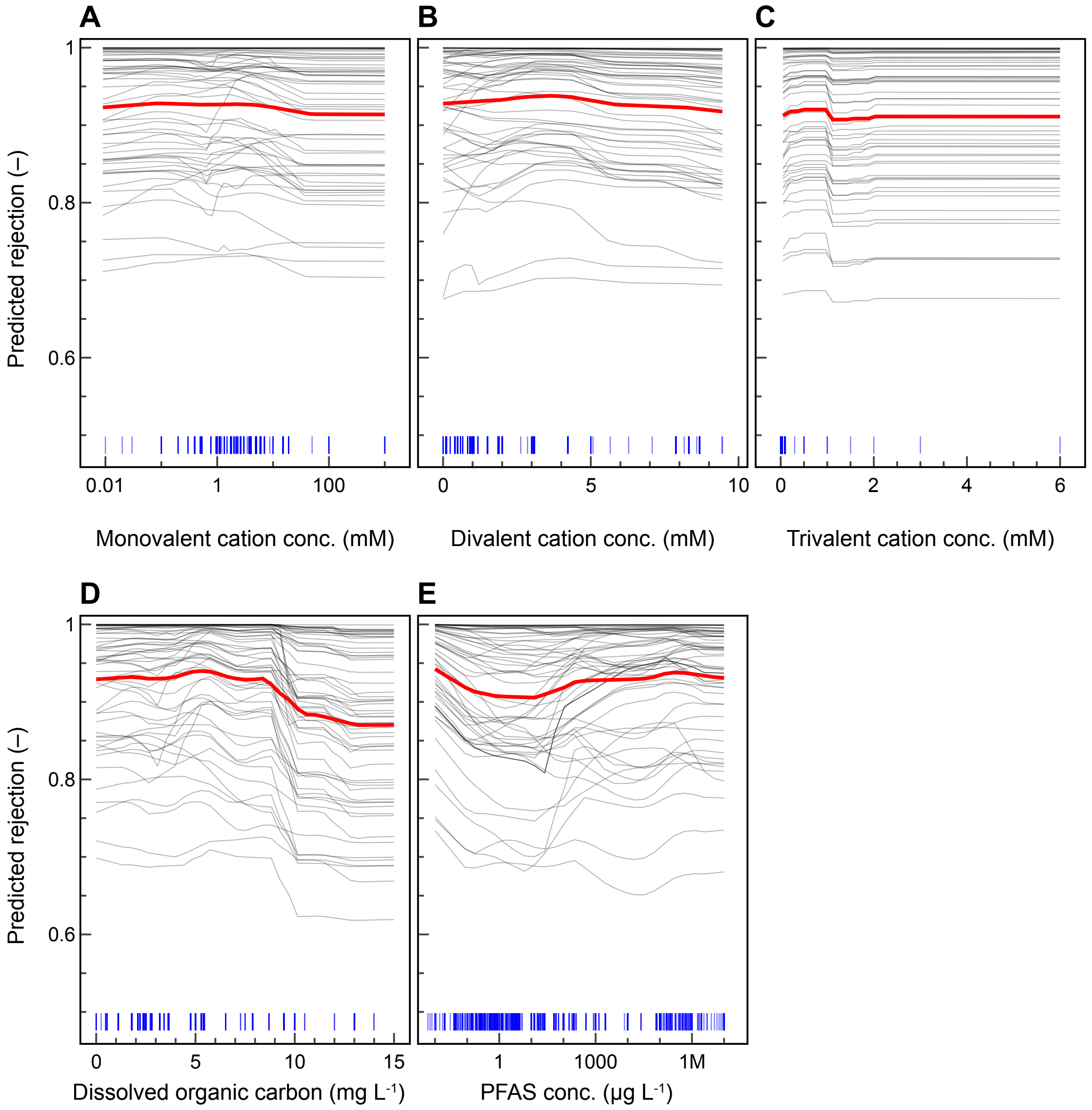

Figure S4. The effect of (a) monovalent cation concentration, (b) divalent cation concentration, (c) trivalent cation concentration, (d) DOC concentration, and (e) initial PFAS feed concentration visualized using ICE plots. Each gray line represents the model response to changes in the respective feature for a randomly sampled set of input parameters while keeping all other features fixed. The red line indicates the average effect (partial dependence) across a random sample of 70 observations, showing the overall trend of how each feature influences the model's predictions. Blue tick marks indicate the distribution of training data values for each feature.

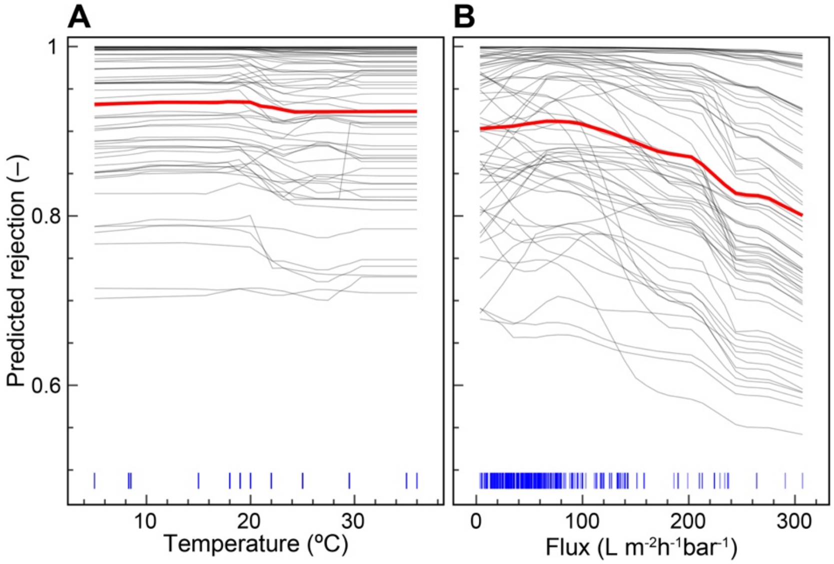

Figure S5. The effect of (a) operating temperature and (b) permeate flux visualized using ICE plots. Each gray line represents the model response to changes in the respective feature for a randomly sampled set of input parameters while keeping all other features fixed. The red line indicates the average effect (partial dependence) across a random sample of 70 observations, showing the overall trend of how each feature influences the model's predictions. Blue tick marks indicate the distribution of training data values for each feature.

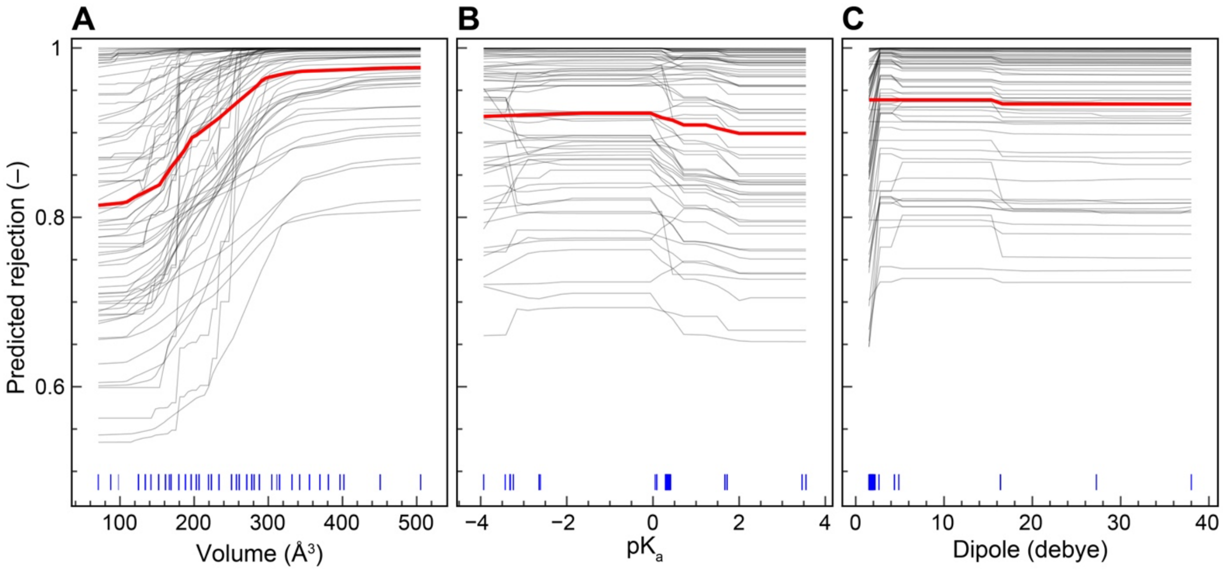

Figure S6. The effect of PFAS (a) molecular volume, (b), pK_a, and (c) dipole moment visualized using ICE plots. Each gray line represents the model response to changes in the respective feature for a randomly sampled set of input parameters while keeping all other features fixed. The red line indicates the average effect (partial dependence) across a random sample of 70 observations, showing the overall trend of how each feature influences the model's predictions. Blue tick marks indicate the distribution of training data values for each feature.

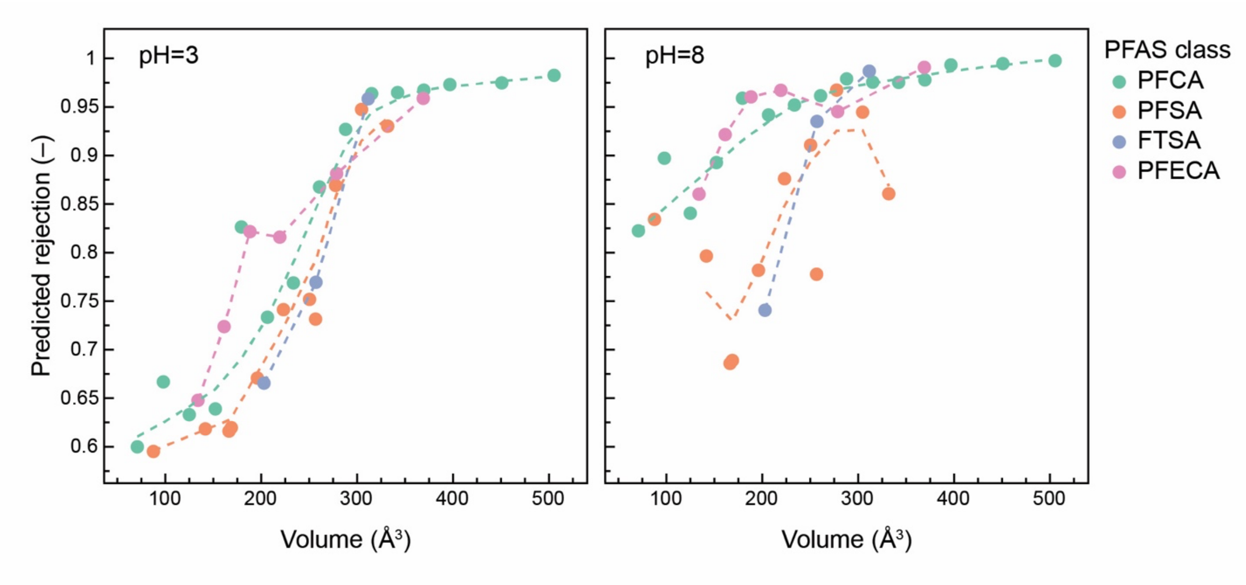

Figure S7. Predicted rejection versus PFAS molecular volume at pH 3 and pH 8 in a pure water feed for a membrane with a pure water permeability of 14 L $m^{-2}h^{-1}bar^{-1}$. Points are colored by PFAS class, with lowess trendlines shown for each class to provide visual guidance. Predictions calculated only for PFAS included in the literature-derived dataset. Variables not specified (e.g. permeate flux, initial PFAS feed concentration) are fixed at the mean of the dataset.
